# Digital Twin for Civil Engineering Systems: An Exploratory Review for Distributed Sensing Updating

**DOI:** 10.3390/s22093168

**Published:** 2022-04-20

**Authors:** Mattia Francesco Bado, Daniel Tonelli, Francesca Poli, Daniele Zonta, Joan Ramon Casas

**Affiliations:** 1 Department of Civil, Environmental and Mechanical Engineering, University of Trento, Via Mesiano, 77, 38123 Trento, Italy; daniel.tonelli@unitn.it (D.T.); francesca.poli-2@unitn.it (F.P.); daniele.zonta@unitn.it (D.Z.); 2 Department of Civil and Environmental Engineering, Technical University of Catalonia (UPC), Jordi Girona 1–3, 08034 Barcelona, Spain; joan.ramon.casas@upc.edu

**Keywords:** digital twin, digitalization, civil engineering, structures, structural health monitoring, SHM, distributed sensing, distributed optical fiber sensors, DOFS, DFOS, review

## Abstract

We live in an environment of ever-growing demand for transport networks, which also have ageing infrastructure. However, it is not feasible to replace all the infrastructural assets that have surpassed their service lives. The commonly established alternative is increasing their durability by means of Structural Health Monitoring (SHM)-based maintenance and serviceability. Amongst the multitude of approaches to SHM, the Digital Twin model is gaining increasing attention. This model is a digital reconstruction (the Digital Twin) of a real-life asset (the Physical Twin) that, in contrast to other digital models, is frequently and automatically updated using data sampled by a sensor network deployed on the latter. This tool can provide infrastructure managers with functionalities to monitor and optimize their asset stock and to make informed and data-based decisions, in the context of day-to-day operative conditions and after extreme events. These data not only include sensor data, but also include regularly revalidated structural reliability indices formulated on the grounds of the frequently updated Digital Twin model. The technology can be even pushed as far as performing structural behavioral predictions and automatically compensating for them. The present exploratory review covers the key Digital Twin aspects—its usefulness, modus operandi, application, etc.—and proves the suitability of Distributed Sensing as its network sensor component.

## 1. Preface

In the past few decades, the rapid progress in information and communication technologies has led to a large-scale integration of computer-aided technologies into the standard practices of almost every industry, including civil engineering and construction. Indeed, acronyms such as CAD, CAE, BIM, FEA, and PDM, are becoming common in the industry and are probably spoken by professionals on a daily basis. Other increasingly trendy and fashionable technologies whose names regularly appear in the context of annual business plans and strategy meetings are machine learning, Big Data, Internet of Things, Digital Twin, artificial intelligence, cloud computing, sensor networks, etc.

All of the above can be summarized in one word: digitalization. This can be defined as the process of collecting information on physical assets and packaging them into a digital representation of these assets that can be processed automatically [1]. As previously mentioned, the adoption of digital technologies in the organization or in the operation environment of a company can be considered a standard and widespread practice at this time. Its impact on modern society has been so radical that some authors have compared it to the 18th century industrial revolution [2,3]. In general, the digitalization of an asset has allowed the automated collection of data that can be subjected to a mining process to better understand process performance, cost drivers, and causes of risk [4]. Problems and risks connected to the operation of these assets can be detected and tackled before they become critical, or bypassed completely due to the possibility of extracting real-time diagnostic data. Buckley and Logan [5] listed the key improvements resulting from digitalization for the process and project outcomes of various industries: fewer errors, greater cost predictability, better understanding of a project, improved scheduling, and optimized design.

For civil engineering systems, digitalization is not only an improvement from an overall design perspective, but also embodies the backbone of the logical next step of the infrastructural management system, i.e., smart infrastructure. Indeed, as society moves deeper into the twenty-first century, the demand for infrastructural assets is growing rapidly, and productivity increasingly depends on these assets [6]. Smart infrastructure is defined as the integration of a sensing network—providing real-time digital information about the state of an asset—with physical infrastructure, in order to achieve real-time monitoring, enhanced service delivery, and efficient decision making for the management of infrastructure assets [7,8]. Additionally, digitalization supports and encourages the convergence of infrastructure networks (enabling coordination between previously uncoordinated activities) for improved efficiency, associated cost reductions, and maintenance [1]. As correctly noted by the Project Digital Built Britain [9], this is all in the name of “enhancing the natural and built environment, thereby driving up commercial competitiveness and productivity as well as quality of life and wellbeing for the public. This will be achieved through better planning, delivery and whole-life management of infrastructure and the wider built environment”. Although the construction industry is currently one of the least digitized sectors, it is predicted that, by 2025, full-scale digitalization will lead to annual global cost savings of 13% to 21% in the design, engineering, and construction phases, and 10% to 17% over the operations phase [10].

The Digital Twin is easily the highest expression of the process of digitalization. Indeed, it not only provides all of the above-mentioned advantages of digitalization and smart infrastructures, but also provides a framework to automate and optimize the “cradle-to-grave” processes associated with operating a civil engineering asset. According to Buckley and Logan [5], although digital tools are utilized in the design stage (36% in USA, 49% in UK, 49% in France, and 44% in Germany), they are not used as much in the construction stage (28% in USA, 7% in UK, 3% in France, and 13% in Germany), and little to no use is made in the post-construction stage (0% in USA, 2% in UK, 1% in France, and 0% in Germany).

The base concept of the present article was born because the authors realized the difficulty of finding clear indications, guidelines, and technical reports on the development of a Digital Twin platform in the literature. Where should one start to develop a Digital Twin? What aspects should be considered? What is the performance required by each component according to its set objective? These are all questions whose answers can only be found in a fragmental and limited way in the existing literature. Furthermore, based on the commonly agreed-upon SHM potential of Distributed Sensing, the authors originally planned to apply these sensors to the Digital Twin for civil engineering systems. However, whenever browsing the literature for studies on the suitability of such a marriage, no material was found. Both of the above-mentioned deficiencies can be explained by the novelty and technological immaturity of the Digital Twin. As such, the present article intends to contribute to the establishment of scientific bases that will enable, in the future, the efficient development of the Digital Twin for civil engineering systems (potentially powered by Distributed Sensing).

The aim of the present article is to examine the key aspects that characterize the Digital Twin concept—its definition, usefulness, modus operandi, applications, etc.—from a civil engineering perspective. Note that this paper is an exploratory review article and not a technical review. As such, the authors do not report specific applications of the Digital Twin to civil engineering systems, but rather collect, define, and expand on the advantages, functioning principles, requirements, and applicative aspects of this implementation. Thus, this paper has a dual purpose: (1) to facilitate an all-inclusive comprehension of the Digital Twin and, consequently, its implementation in industry and research; and (2) to assess the potential of Distributed Sensing as a key component of the Digital Twin. Regarding the former, the reader should keep in mind that, at present, the implementation of the Digital Twin in civil engineering systems is still in its infancy; so much so, that very few applications can be found in real life. Finally, a review of actual real-world applications of the Digital Twin in civil engineering systems will be the focus of the authors’ subsequent publication.

In this paper, the discussion on the key principle of the Digital Twin–civil engineering systems marriage is developed around the traditional “What, Why, How, Who and When” approach. This ensures a comprehensive vision of all of the multiple facets of the subject in question. In particular, after *Section 2. Introduction*, *Section 3. Digital Twin: its multidisciplinary potential and applications* tackles the first two questions in a universal manner nonexclusive to the civil engineering field. Indeed, *Section 3.1. What Are Digital Twins?* will answer the “what?” question by providing the reader with a formal but informative definition of the Digital Twin. *Section 3.2. Where Digital Twins?* will answer the “where” question by providing the reader with an overarching perspective of modern-day Digital Twin applications in both research and industry. *Section 4. Towards the Digital Twin for Civil Engineering Systems* moves the dialogue towards the civil engineering field. *Section 4.1. Why Digital Twins?* answers the “why?” question and, in particular, why infrastructure managers and stakeholders should integrate the Digital Twin concept into their infrastructural stock. *Section 4.2. How Digital Twins?* delves into the technological aspects unique to the development of a Digital Twin for a civil engineering system i.e., “How [to develop them]?”. Finally, *Section 5. Distributed Sensing as Digital Twin Sensor Network Component* presents the advantages of a combination of these two cutting-edge technologies. *Section 6. Conclusions* summarize the key points extracted in Section 4 and Section 5.

## 2. Introduction

We live in an environment of ever-growing demand for transport infrastructure networks, because these are relied on for the functioning and growth of regional economies. Unfortunately, we also live in an environment of ageing infrastructure. Most bridges, for instance, are reaching the end of their service lives and are generally inadequate to cope with the increasing traffic demands and resilience requirements. Revealing data, which are, on average, representative of the situation of most countries worldwide, are provided by the American Society of Civil Engineers (ASCE)’s 2021 Infrastructure Report Card [11]. According to this report, of all the bridges in the USA, 42% are 50 years old or more and 7.5% are structurally deficient. On average, there are 178 million trips across a structurally deficient bridge each day. Regrettably, this situation has led to an increasing number of structural failures of highly populated infrastructural assets such as residential buildings and bridges. In just the decade from 2011 to 2021, around 60 bridge failures occurred worldwide, followed by an equally large number of fatalities. Of these failures, a conspicuous example was the sudden and deadly collapse of the Morandi Bridge in Genova (Italy) in 2018, which killed 43 people. For comparison purposes, the previous decade saw the collapse of around 50 bridges; the decade prior to that saw 15; and the decade prior to that saw 20.

However, as observed by Regier and Hoult [12], it is not feasible to replace all of the structures that have surpassed their intended service lives, because of budgetary, logistical, and environmental concerns. The only other possible approach consists of keeping the assets that are still fit for purpose in service. As part of this new global and national effort, engineering enterprises worldwide are required to embrace both classic and novel methodologies to ensure the durability of national infrastructural assets and to reduce the processes of rehabilitation, restoration, and regeneration.

The best approach to the issue of infrastructural durability is universally considered to be Structural Health Monitoring (SHM) [13]. SHM can be understood as the continuous measurement and analysis of key structural and environmental parameters under operating conditions, for the purpose of warning of abnormal states or accidents at an early stage [14]. Indeed, accurate information from the monitoring of structures is crucial to make the right decisions on maintenance and user safety. However, these two aspects do not wholly describe the potential of SHM. Indeed, as a by-product of keeping old infrastructure in service, new environmentally taxing constructions that are not strictly necessary can be avoided. Therefore, an environmental and sustainability potential is also embodied by SHM. However, SHM, together with its intrinsic potential for sustainability, is still a young scientific practice. As such, despite the increasingly large number of SHM-oriented applications of condition-assessment inspections, testing, and monitoring, a scientific consensus on their implementation is still absent. Consequently, to this day, such practices have not been adequately addressed in structural codes and standards, leading to different technological interpretations and implementations in every country. This lack has been acknowledged by both scientific and governmental institutions. For instance, it is the focus of the European-wide “IM-SAFE project” [15], whose main objectives are to clarify and integrate the monitoring and maintenance-related “rules in the structural design codes by filling-in the gaps in the current knowledge and closing the gap between the standard and the practice [15]”. In other words, IM-SAFE plans to support the European Commission and the European Committee for Standardization in preparing new standards in monitoring, maintenance, and safety of transport infrastructure.

One of the greater challenges facing the civil engineering industry (formerly known as the so-called “brick and mortar” industry) and, therefore, SHM, is adapting in record time to the “digital push” and the consequent movement towards a full digitalization of the existent infrastructural stock. This, for example, is the idea behind Building Information Modeling (BIM) which, according to ISO 19650:2019, is the use of a digital representation of a built asset to facilitate design and construction, and to form a reliable basis for decisions. One can immediately see “the potential of BIM for the management of all the information and documentation of an infrastructure project throughout its life cycle in a digital environment” [16]. The previous wording of “throughout its life cycle” is key, as it suggests how digitalization can help to boost the durability of the existent infrastructure even as it reaches the end of its service life. To meet and exceed an infrastructure’s intended purpose, a parallel intelligent digital platform has been very recently introduced, i.e., the Digital Twin.

As amply explained later, the Digital Twin concept, similarly to BIM, also uses a digital reconstruction of a real-life infrastructure or building, but takes it one step further by integrating the former with data produced by sensors positioned on the latter. This sensor deployment effectively acts as the nervous system of the real-life structure by providing information on its well-being (structural health) or issues. A constant and automatic data interchange (deformation, temperature, occurrence of hazardous events, etc.) between the real-life and digital structure is therefore created. Through the integration of these sensors in the so-called “smart buildings” or “smart infrastructure”, and through their respective Digital Twin, concessionaries, stake holders, managers, and decision makers can take data-based, informed, and “smart” decisions to ensure the durability and safety of their infrastructural stock. This is perfectly embodied in Bolton et al. [9]’s Gemini Principle statement: “Digital twins of physical assets are helping organizations to make better-informed decisions, leading to improved outcomes”.

Overall, the global Digital Twin market size was valued at USD 3.1 billion in 2020 and is projected to reach USD 48.2 billion by 2026 [17]. In regards to the construction industry, it is estimated that the integration of Digital Twins will allow for savings of 15–25% by 2025 [18].

As can be determined from these numbers, the potential of a Digital Twin integration into civil engineering systems is becoming increasingly apparent and sought after. An example of this is Europe’s novel “ASHVIN: Digitizing and transforming the European construction industry” project [10]. This is a joint effort among 14 European partners that has the final goal of proposing a European-wide Digital Twin standard, an open-source Digital Twin platform integrating the Internet of Things, image technologies, and a set of tools/procedures to apply the platform, in addition to the standard proven to ensure specified productivity, cost, and safety improvements [10]. Interestingly, ASHVIN will demonstrate its innovations on ten real-world projects across Europe and different areas of construction, including three different railway bridges in Plasencia-Bajadoz (Spain), an airport runway in Zadar (Croatia), and office buildings in both Göteborg (Sweden) and Barcelona (Spain). Furthermore, as explored later, ASHVIN’s main page [10] conveys another large potential of the Digital Twin, namely, the use of the constant flow of real-time data between the twins and the historical data from other projects to provide the required historical foundations for predictive and prescriptive analytics.

The aim of the present article is not only to explore the potential benefits of a Digital Twin integration into civil engineering systems, but also to assess the suitability and efficacy of the Distributed Sensing monitoring tool as the “sensor deployment” tasked with feeding data into the Digital Twin. The reader should know that, whenever referring to monitoring tools, one generally refers to instruments falling under the category of “traditional monitoring techniques”, namely, inclinometers, accelerometers, extensometers, total station surveys, load cells, GNSS-based sensors, etc. [19,20]. By themselves, these can be considered sufficiently reliable since their correct deployment has been extensively investigated and they are now widely acknowledged, thus ensuring reliable monitoring and structural assessments. However, as stated in Baker [21], conventional forms of inspection and monitoring are only as good as their ability to uncover potential issues in an accurate and timely manner. Indeed, regarding the ability of damage detection, traditional tools present several drawbacks, which include the need for the infrastructure’s service interruption during their deployment; non-automated, non-real-time measurements; and interference risk.

**Figure 1:**
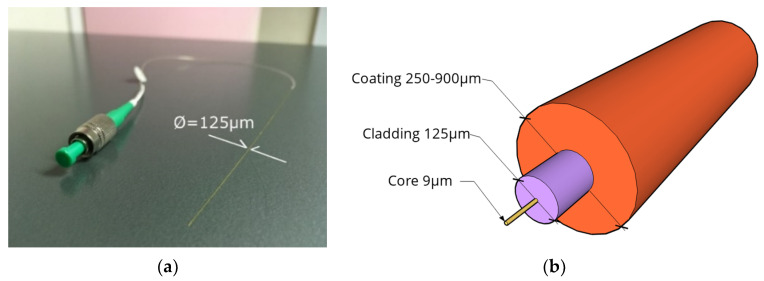
Optical Fiber Sensors: (**a**) a picture of the fiber and a (**b**) 3D illustration of its cross-section—note that the latter is of an indicative nature only as many kinds of differently coated OFS are available on the market [19].

Modern monitoring technologies, instead, are aimed at tackling these limitations (fully or partially surpassing them, depending on the sensor) by boosting the sensors’ precision, automation, ease of deployment, etc. These technologies include, among others, Optical Fiber Sensors (OFS), Global Positioning Systems (GPS), radars, Micro Electro Mechanical Systems (MEMS), and Image Processing Techniques such as Digital Image Correlation (DIC) [20,22]. In particular, OFS are dielectric devices used to confine and guide light, and consist of several layers: a fiber core, cladding, and, occasionally, an external jacket aimed at providing the fiber with mechanical resistance (see Figure 1).

A significant ability of OFS is the measurement of mechanical and temperature-variation-induced strains along the fiber length by means of light scattering and back-scattering, which occur whenever the photons of the emitted light interact with the physical medium through which it travels (the fiber’s core itself). When no strain or temperature is imposed on the system, light propagates and is reflected throughout the imperfections with a given signature. By comparison, when strain or temperature values vary, the frequency of the back scattered light is shifted. The measured strain values are then related to the frequency shift. As a matter of fact, three different types of light scattering and backscattering may occur in an OFS, namely, Raman, Brillouin, and Rayleigh [23]. All have particular optical features that make one more suitable than the others relative to the research objectives. Rayleigh backscattering, for example, allows for particularly accurate strain sampling despite its reduced sensing range limit. This kind of backscattering represents the working principle behind Distributed Optical Fiber Sensors (DOFS or DFOS) or Distributed Sensing technology.

Distributed Sensing utilizes OFS and, most importantly, an interrogator machine able to accurately measure strains (down to 1 µε), temperature, and vibration in two or even three dimensions [24]. These measurements can all be achieved in a completely distributed manner (modern interrogation units can attain a spatial resolution of 0.63 mm) and with measurement frequencies of 250 Hz [25]. Consequently, direct detection and characterization (including recognition, localization, and quantification or rating) of local strain changes generated by structural damage are intrinsic properties of such sensors. Furthermore, Optical Fiber Sensors have some inherent advantages, such as corrosion immunity, high durability, resistance to electromagnetic interference, small size, and light weight, that elevate them beyond the classic monitoring tools [26]. On the basis of all of the above characteristics of Distributed Sensing, the potential of this monitoring tool for SHM applications becomes quite evident.

In fact, DOFS have already been employed in numerous real-life SHM applications, including the monitoring of buildings (including skyscrapers), bridges, roads, geotechnical engineering applications (pile foundations, soil and rock deformations, soil stabilization anchors, mining), tunnels, pipes, and wind turbines. The authors redirect those readers interested in a general overview of all of the above applications to the following literature reviews: Bado and Casas [19] and Barrias et al. [23].

As previously noted, the present article first presents a short exploratory review of Digital Twins, how they work, and the advantages of their application to the civil engineering field. Finally, given the suitability of Distributed Sensing to SHM purposes, this paper attempts to assess whether the performance of this tool meets the recommended requirements for its implementation in a Digital Twin framework.

To properly conclude this introduction, the authors iterate a concept originally reported in Seo [27], namely, civil engineering’s slow transition towards technology convergence. This is a theory that asserts the absence of a clear distinctive line between the products and applications of different disciplines; instead, a co-existence, intersection, and reciprocal enhancement exist between the latter which, in turn, accelerates the overall technological advancement [28]. The application of the Digital Twin to the civil engineering field and its integration with Distributed Sensing monitoring technology to create “smart” structures is the quintessential representation of such a development, as it joins civil engineering, photonics, signal processing, materials engineering, computer engineering, etc.

## 3. Digital Twin: Its Multidisciplinary Potential and Applications

In order to gain a clear and general picture of the Digital Twin concept, the authors dedicate the present section to understanding exactly what Digital Twins are and the degree to which they can be applied to multidisciplinary challenges.

### 3.1. What Are Digital Twins?

Although there seems to be no general consensus on the origin of the Digital Twin, two citations regularly appear whenever attempts are made to identify its source, which have unofficially become the origins of this concept. These are (1) a presentation held at the University of Michigan on Product Lifecycle Management by Grieves in 2002 [29], and (2) a strategic technology roadmap published by NASA in 2010 [30].

Regarding a formal and unique definition of the Digital Twin, due to its recent emergence—as seen, the concept is only two decades old—a common agreement has also not been reached. The definitions often seem to be interconnected to the specific end goal of each Digital Twin application and, thus, vary slightly depending on the case. As part of the review effort, the authors of the present article consulted and analyzed numerous Digital Twin definitions from both frequently cited and novel scientific publications [31,32,33,34], and have reached a unanimous agreement on two.

The first of these is a simpler and concise definition that nevertheless manages to convey the core concepts behind a Digital Twins. The second is a less straightforward but more technical definition that not only elucidates the meaning of Digital Twin but also paints a clear picture of its key features. The authors decided to report only two definitions of the Digital Twin concept, instead of the many available definitions, to promote the convergence towards a single formal definition. Furthermore, the authors call for larger efforts—from both industry and academia—to achieve this important goal. Reaching this goal could represent the first step towards uniform methods, standards, and norms for the creation, employment, and optimization of Digital Twins.

The first definition is from Boschert et al. [31]: “*A [digital] comprehensive physical and functional description of a component, product or system together with all available operational data […] useful in all the current and subsequent lifecycle phases*”.

The second definition comes from Defraeye et al. [32]: “*A Digital Twin of a certain product is defined as a virtual representation of its real-world counterpart, which (1) contains all essential elements, such as all geometrical components and material properties; (2) simulates accurately and realistically all relevant processes and their kinetics throughout the product’s life-cycle; and (3) is connected to the real-world product and processes by sensor data, which is preferably continuously updated in real-time*”.

These two definitions relay all of the key information on what a Digital Twin is:

- Digital Twins are virtual representations or replicas of a physical real-world asset (that is, the Physical Twin). This can be achieved though 3D modeling, finite elements, etc. Note that there is practically no limitation to what this real-world asset can be—a manufacturing product [35], a horticulture product [32], any man-made asset of the built environment [1], etc.
- In contrast to a traditional digital model (developed, for example, by means of Computer-Aided Design software), Digital Twins are not restricted to being just geometrically accurate digital replicas. Instead, as correctly stated in Lu and Brilakis [18], in a Digital Twin, the geometric and graphical data are enriched by semantic information, i.e., massive, cumulative, real-time, real-world data measurements. These data include, but are not restricted to, engineering data (movements, deformations, overheating), operational data, behavior descriptions, inspection reports, and maintenance history. As explained below, there additional data are extracted from the Physical Twin by means of sensor networks—sensors, Internet of Things components, etc.—positioned on it and paired or “twinned” to its Digital Twin by means of a software framework. By twinning the Digital Twin with the Physical Twin asset—via streams of real-time data from the embedded sensors—one can visualize live data that describe the real-life asset, execute contextual inquiries, and perform any sort of data exploration [36].
- The third and last key piece of information relayed by the two above definitions is the utility of the Digital Twin throughout the life cycle of the real system. Indeed, as constant updated input data are fed to the Digital Twin, it evolves alongside its Physical Twin, constantly reporting and updating the currently available knowledge about the static and dynamic status of the analyzed system. This last aspect is crucial to make informed management decisions and implement novel solutions across the various life cycle phases of a system. This represents a departure from the commonplace practice of treating the design and operation of a system as separate phases.

Note that, in the Digital Twin literature, a distinction between the Digital Twin and the Digital Shadow is sometimes made [1,32,33,37] as follows: a Digital Shadow offers a one-way data flow from the physical to the digital object, whereas a Digital Twin embraces data exchange in both directions between the physical and digital assets.

### 3.2. Where Digital Twins?

Digital Twins are applied in a wide range of fields, some of which are niche and very specialized, but most of which are conspicuous and present in our daily lives.

Among the former, it is worth mentioning what is considered the earliest application of Digital Twin, even before it was called such [38]. In the 1970s, when planning the Apollo 13 space mission, NASA employed a “simulator” concept intended to reflect the condition of the spacecraft. On the day of the launch (11 April 1970), an explosion in the oxygen tanks critically damaged the main engine of the service module, causing oxygen to leak into space. Although Apollo 13 was obviously not equipped with technology able to collect data on the state of the module’s components and beam them back to base, NASA used its state-of-the-art telecommunications technology to stay in touch with its spacecraft and modify the simulators in order to reflect the real-life condition of the crippled craft [38]. Ultimately, the crew returned to Earth safely. NASA now uses the Digital Twin for both manned and unmanned aircraft [32,39].

Regarding more “down to Earth” applications, Digital Twins are now being developed for entire production plants—for the manufactured products on the production line and for the machines building it [32]. TESLA, for example, aims to develop a Digital Twin for every built car and, by means of the consequent synchronous data transmission between the car and the factory, provide maintenance schedules tailored individually to each user, thus optimizing resources [40]. Energy companies such as General Electric and Chevron, in an effort to forecast the health and performance of their products over their lifetime, use Digital Twins to track the operations of wind turbines [39]. Yet more wide-ranging applications are regularly being proposed or are even already in use. Defraeye et al. [32] studied the possible application of the Digital Twin to the supply chain of fresh horticultural products. The authors highlighted the potential of monitoring the history of each shipment because it is exposed to a unique and unpredictable set of temperatures and gases as it is transported from the farm to the consumer. Saifutdinov et al. [41] describe the application of a Digital Twin to an airport centralized traffic control system that can be trained to perform the controller’s tasks through the implementation of machine learning. In Granacher et al. [42], the authors propose the use of a Digital Twin to assist decision makers, steering their exploration of the multi-criteria solution space and guiding them towards the most optimal decisions, independently from the instance in which their choices are inputted into the system. On the other side of the spectrum, Quilodrán-Casas et al. [43] used the functionalities of the Digital Twin to develop epidemiological models aimed at gaining a better understanding the spread of the COVID-19 disease. In particular, to study the dynamics of epidemiological models, the authors used official virus spreading data from the UK to developed two different Digital Twins of an idealized town, taking account of the spatial dissimilarities.

As previously noted, among the most ambitious and complex applications of the Digital Twin technology are “Smart Cities”. Assuming that understanding the “urban metabolism” of a city (the production, import, and export of diverse natural and non-renewable material flows) is key to reaching the goal of a sustainable city, the Smart City technology powered by the Digital Twin is an ideal candidate to achieve this goal [44]. The whole idea uses the Digital Twin as a representation of a city in terms of its physical assets [45], such as its geography/topography, energy and consumption, traffic, infrastructure, public safety, transportation, environmental sanitation, social services, health and hygiene, culture and tourism, parks and entertainment, and water resources [46]. Ultimately, a Smart City is a large-scale Digital Twin expressly designed with a people-centered perspective, thus providing solutions aimed at improving the citizens’ quality of life from a holistic point of view, i.e., comprehensively encompassing all of its relevant aspects, such as the economy, society, and environment [1]. It is acknowledged that the Digital Twin of a Smart City should ideally provide a real-time response to the diversified needs of its residents [46]. Its advantages, though, are not restricted to a reactive operational framework (i.e., operational decisions taken “in response” to data reported by the sensors deployed in the city), but can be also of a preventive nature. Indeed, the Digital Twin of a Smart City enables urban planners, engineers, and architects to simulate solutions to the city’s problems without actually implementing them, a practice named in [47] as Virtual Experimentation and Virtual Test-Bedding. This kind of research can cover a vast number of both independent and intertwined aspects of life in a Smart City, from mobility with connected and autonomous vehicles, and health care in cyberspace [48], to optimization solutions such as the integration of Deep Learning in a Smart City’s Digital Twin powered by both Internet of Things components and Big Data Analysis technology [46]. To provide an idea of the evolving popularity of the Smart City technology, the global revenue for Smart City technologies, products, and services is projected to be worth around 150 billion U.S. dollars in 2022 and around 250 by 2025 [49].

Some applications and prototypes of the Digital Twin technology in favor of a Smart City can be found in Helsinki (Finland) [44] and Herrenberg (Germany) [50], but the most well-known integration is Virtual Singapore [47]. The Singaporean Government, in collaboration with Dassault Systèmes [51], has created a three-dimensional model supported by static and dynamic data from a pervasive set of sensors acting as a collaborative data platform for the city. As stated in Dassault Systèmes Virtual Singapore dedicated page [20]: “As part of its Smart Nation effort, Singapore wanted to develop a Smart City environment to plan everything—from emergency evacuation to comfortable urban living”. The city has already taken advantage of this technology. As example, in an attempt to manage and contain the COVID-19 virus, in April 2020, the Singaporean Government launched a platform named Space Out [52], which displays the city’s Digital Twin data about crowd levels, thus enabling its citizens to make informed decisions in regards to safe distancing. The city’s Digital Twin was also developed with the scope of enabling the test-bedding of novel concepts and services, planning and decision making, and research on emerging technologies. Some examples of this kind of application are:

- Calculating the amount of energy that could be generated by installing solar panels on certain roofs;
- Developing and optimizing evacuation models for disaster management;
- Visualizing existing the landscape against ongoing and future construction projects to harmonize and promote holistic urban planning;
- Identifying barrier-free routes for the disabled and elderly, taking into consideration the surrounding physical landscape barriers, i.e., water bodies, vegetation, and infrastructure.

**Figure 2:**
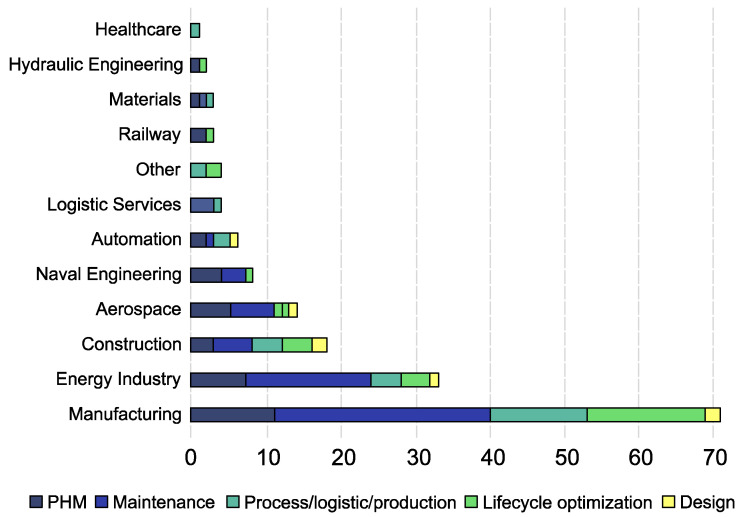
Distribution of publications on the Digital Twin across various fields, where PHM stands for Prognostic Health Management [53].

To gain a general perspective on the distribution of Digital Twin applications over the various scientific domains, one can check the number of scientific publications per field. Figure 2 shows a graph from Errandonea et al. [53] displaying the distribution of publications on Digital Twins across various fields from 68 different journal articles, conference proceedings, book chapters, reviews, and business articles collected from the “Scopus” and “Web of Science” databases. As shown, the manufacturing and production disciplines encompass the bulk of Digital Twin-focused scientific publications [34,53]. The construction field represents roughly one-third of the publications. Indeed, although the concept of the Digital Twin is increasingly gaining attention and relevance in the field of civil engineering and in the management of the built environment, it is far from reaching sufficient technological maturity [54]. This deficiency is one of the key reasons why infrastructure managers are reluctant to undertake the economically and logistically challenging procedure of implementing this innovative technology.

## 4. Towards the Digital Twin for Civil Engineering Systems

The previous section focused on providing the reader with a general and multidisciplinary perspective on the definition of Digital Twins and the breadth of the spectrum of their possible applications. The present section restricts the discussion to civil engineering systems and, in particular, on the advantages provided by integrating these systems with Digital Twins. This section also explores the practical and technical aspects to be considered when attempting such an implementation.

### 4.1. Why Digital Twins?

Because civil engineering infrastructures cover key roles in the proper functioning, security, and comfort of modern society, their time-induced deterioration and issues that jeopardize their serviceability (e.g., corrosion, fatigue, creep, and shrinkage-induced shortening and cracking) should be treated with equivalent criticality.

The current maintenance, rehabilitation, and retrofitting approach to civil engineering systems is mostly time based (highway bridges and railway tunnels are typically inspected once a year [55]). Generally speaking, the effectiveness of a maintenance approach is only as good as its ability to detect in a timely manner the surge in criticalities and damage to the structure [56]. Unfortunately, regarding the time-based approach, these issues can only be detected during an inspection and at no other time. This delayed detection, and the consequent delayed maintenance intervention, exposes the infrastructure users and managers to an increased risk of structural failure. Although more frequent inspections may seemingly solve this problem, more often than not they do not happen in light of the involved operational costs and larger infrastructure downtime. Given these downsides of the time-based approach, some authors called for an evolution of this maintenance philosophy into a potentially more cost-effective condition-based approach [57]. According to this approach, managers and operators can optimize the allocation of their budget by performing structure maintenance only on those assets that actually require it, when they require it. According to Farrar and Worden [57], the key prerequisite of the condition-based maintenance is the deployment of a sensing system on the infrastructural assets. This would then be able to monitor their response, notify the operator of the emergence of defects or damage, and allow for corrective actions to be taken in a timely fashion, well before the damage evolves into failure.

Condition-based monitoring, just like time-based monitoring, pertains to the category of reactive systems, as opposed to preventive systems (i.e., systems able to anticipate and prevent potential hazards) [1]. If the adoption of the latter became common practice among infrastructure managers, the operational life of civil engineering systems could be significantly extended [57]. Not only can the Digital Twin concept help to establish the condition-based maintenance approach as standard practice, it can also expand the concept’s potential further by enabling fully preventive approaches.

**Figure 3:**
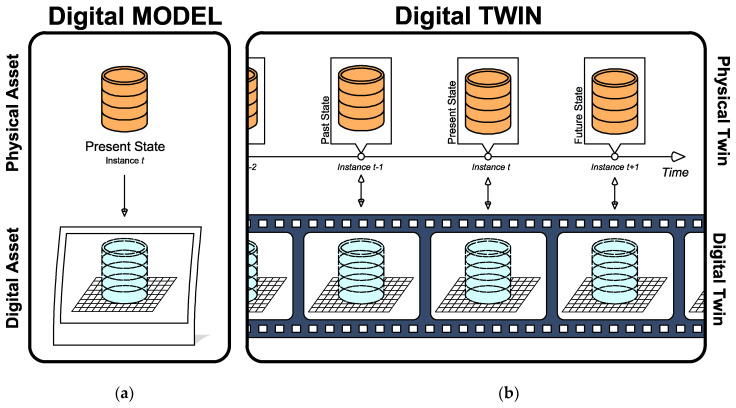
The output of (**a**) a digital model (CAD or BIM) analogous to a “picture” of the asset at the instance it was modeled and (**b**) a Digital Twin analogous to a “set of pictures” or “video” of the evolution of the asset over time.

Before describing how this can be achieved and how the Digital Twin can be of great use for optimal management of civil engineering systems, it is important to look back to its predecessor, i.e., Building Information Modeling (BIM). As defined by Camposano et al. [54], BIM: (1) formally comprises a wide range of information systems used to generate, control, and manage building information; and (2) supports the representation of built assets—in terms of their 3D geometry and functional attributes—and their relationships using structured interoperable instruments. Furthermore, according to ISO 19650:2019, BIM allows for the use of a digital representation of a built asset to facilitate design and construction, and to form a reliable basis for decisions. Its introduction in the early 2000s allowed practitioners and researchers to represent not only the geometric characteristics of the components of an asset (previously made through Computer-Aided Design or CAD), but also their interconnections. It is acknowledged that models based on CAD and, subsequently, on BIM, describe an asset in increasingly higher detail. However, it is important to understand that these models are only representative of a particular asset at a specific instance in time. In other words, whenever one develops a BIM model of an asset, it is analogous to saying that one is “taking a picture” of that asset just as it is at that specific moment (see Figure 3a).

Digital structural models are often used for the design of a structural asset. Such models can be compared to a “blurred picture” of the structure because the “a priori” nature of its input parameters (i.e., without any evidence from the realized structure, only according to design codes) introduces a relevant degree of uncertainty in their design and construction processes. Only once the structure has been constructed can the design parameters be fully determined—and the uncertainty eliminated—through Non-Destructive Tests (NDTs) on material samples (e.g., pull out tests) and on structural elements (e.g., proof load tests) [58]. Continuing the “picture” analogy, if one re-entered the above-mentioned test results into the original design model (in other words, updating it “a posteriori”), one would obtain a closer and less blurred “picture” of the realized asset. Although this “picture” is highly detailed—even going as far as to describe the innermost connections between the various parts of its subject—it is nevertheless only a representation of the asset at the instance that the “picture” was taken.

Now, assuming that the modeled subject (aka the subject of the “picture”) is a civil engineering infrastructure asset—a bridge for example—it is of paramount importance to consider its structural health and condition during the entirety of its service life, and not only at a single instance (i.e., the moment the “picture” was taken). Therefore, considering that any structural system undergoes some degree of deterioration over time due to natural aging and service operation (the main concerns in roadway bridges are fatigue and corrosion), it is important to keep structural models updated to allow them to be used for operational decision making. Furthermore, given a specific infrastructural asset, the models should accurately represent the evolution of the asset-specific aging symptoms in its asset-specific environmental context [58]. However, here is precisely where the setup of traditional structural models fails and that of Digital Twins has significant potential.

As noted earlier, the trait of the Digital Twin that best embodies its potential is precisely its continuous updating of the virtual model, which therefore “evolves” in conjunction with the Physical Twin. This means that the virtual model progressively and automatically integrates the deterioration of the real-life asset. As such, Digital Twins, in contrast to standard virtual models, exist as a “sequence of pictures over time” or as a “video” (see Figure 3b), with a refresh rate equal to the updating frequency of the structural model. Due to its ability to track the life cycle of a physical asset across various points in time, the Digital Twin can critically support decision making in both the design phase of a civil engineering structure and in its operational life.

Having clarified this general framework, we now delve deeper into why infrastructure managers should implement Digital Twins into their infrastructural stock. The following discussion is separated into several points as follows.

- Damage detection efficiency. As specified by Gunner et al. [59], mathematical models have long been used to identify the surge of criticalities in civil engineering structures, but “comparison with recorded measurements is traditionally done in a one-off model validation exercise”. This means that structural criticalities can go unnoticed, thus representing a risk for the users and extra costs for the infrastructure managers due to delayed interventions. Conversely, continuously monitoring of the response of a structural system allows a Digital Twin to frequently—and automatically—update and revalidate its model. This, in turn, provides visibility to the evolution of key structural parameters and permits their continuous comparison with their predicted counterparts (calculated within the digital model). Abnormal deviations between the two, which are typically a symptom of structural damage [60] (e.g., the failure of one or more pre-stressing tendons [61]), can therefore be swiftly identified and a timely response triggered.
- Decision making support. The Digital Twin provides infrastructure managers and decision makers with functionalities for controlling, monitoring, and optimizing a physical asset. In those situations where maintenance interventions are required, the Digital Twin allows for their proper timing and prioritization through data-driven, updated, and accurate procedures [31]. Most importantly, however, the Digital Twin supports decision makers by providing information on the structural reliability of an asset, whether under daily operative conditions or after extreme events such as earthquakes and floods. Based on this information, and with the help of predetermined thresholds and emergency plans, the Digital Twin can help decision makers face emergency situations, thus enabling swift and efficient responses. Considering all of these aspects, it is easy to imagine how Digital Twins can be integrated into a holistic Decision Support System for infrastructure managers.
- Addressing the infrastructure managers’ skepticism about SHM. It should be mentioned that infrastructure managers have a degree of skepticism about the trustworthiness and effectiveness of SHM as an instrument on which to base important operational decisions. As a matter of fact, it is not uncommon that, despite the presence of a SHM system in place, infrastructure managers still make decisions driven solely by their experience or simply by common sense [62]. From this point of view, Digital Twins may be a turning point for the industry because they have the potential to bridge the gap between the technical sensing system/signal processing world and the managerial world. Indeed, through its synthetic and graphic representational format, a Digital Twin can finally allow infrastructure managers to clearly and intuitively visualize and interpret the monitoring data, comprehend the current behavior of a structure, and choose the best management strategy accordingly. In summary, when combining the Digital Twin’s intrinsic potential for automated damage detection, maintenance planning, and emergency response, with its intuitive representation of monitoring data, it is finally possible to eliminate the skepticism of infrastructural managers in regard to SHM.
- Predictive maintenance approach. The Digital Twin allows for behavioral predictions of an infrastructural asset that can be used to understand ahead of time whether the structure may transit towards a damaged state (e.g., cracking) or exceed the value for its serviceability limit (e.g., excessive deflection). The forecasting of future courses of action can be expanded by incorporating simulation models, data analytics, or machine learning features [54]. For example, by monitoring localized structural failures over long periods of time—for instance, the opening of cracks in a reinforced concrete element [63]—machine learning algorithms can track key performance indicators so that failure modes can be predicted and maintenance planned [36].
- Potential for automation of infrastructure. A Digital Twin can potentially link both the Physical and the Digital Twins with a bi-lateral data exchange. Through such a data feedback loop, both simulated and real twins can develop capacities for autonomy and “learn from and reason about their environment” [64].
- Potential for sustainability. As a Digital Twin evolves over time with the constant input data fed into its system, the “right-time interventions” empowered by this technology can not only expand the service life of the built infrastructure, but also lead to a reduction in emissions and in the modern day redundant use of raw material [65]. Therefore, the implementation of Digital Twins translates into increased efficiency, sustainability, and resilience of civil engineering systems [1].

**Figure 4:**
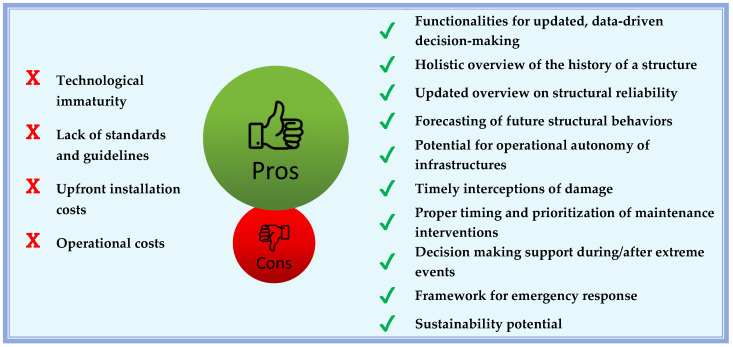
Pros and cons of a Digital Twin implementation in a civil engineering system from an infrastructure manager perspective.

The authors believe that to properly end the present section it would be suitable to summarize the advantages and disadvantages of a possible Digital Twin implementation in a civil engineering system, as shown in Figure 4.

### 4.2. How Digital Twins?

When designing a Digital Twin, several key aspects need to be taken into consideration, such as: the choice of digital modeling technology, software framework, sensor network, sensor positioning, data input refresh rate, and operational thresholds. However, before considering any of these aspects, one needs to clarify and outline the End-User Requirements (EURs) of the Digital Twin.

Defining the EURs is equivalent to asking: “what kind of data do I want the Digital Twin to collect and report”? This is not always obvious. Tao and Qi [39] exemplify how complicated this can be: “To model a wind turbine, for example, [one] might require [the] monitoring of vibrations from the gearbox, generator, blades, shafts and tower, as well as of voltages from the control system. Torques and rotation rates, temperatures of components and the state of the lubricating oil must also be tracked, together with environmental conditions (wind speed, wind direction, temperature, humidity and pressure)”. Generally, the EURs of a Digital Twin define the information required by its end-users [18]—whether these are engineers, infrastructure managers, both, or others—or, that is, the expected output for the proper design/management of the asset in question.

For this purpose, defining the EURs requires a profound knowledge of the asset under analysis and of all the external factors that might affect its performance. In [59], Gunner et al. accurately defined the various associated with EURs, from their definition to their practical outcomes; therefore, the authors of the present article encourage consultation with this reference. In order to define the EURs of a Digital Twin, one needs to: (1) identify all the possible risks to the structural integrity of the asset and to the safety of its users; (2) establish what data is required in order to capture the presence of such a risk; (3) identify possible actions, interventions, and procedures to mitigate these risks; and (4) establish what data is required in order to properly plan and put into practice these mitigation actions. It is through the extraction of this information (how to identify and mitigate the risk) that a Digital Twin can efficiently assist decision makers in their efforts to extend the service lives of their infrastructure. Note that, in most situations, decisions such as those above are multifactorial, i.e., they depend not just on the state of the structure, but also on other aspects such as the availability of resources [66]. Therefore, the greater the number of influencing factors visible to the Digital Twin, the more appropriate its suggested course of action. Once the EURs are defined, the actual design of the Digital Twin can finally take place. The following step concerns the virtual model itself.

Generally, for civil engineering applications, virtual models of an asset are developed by means of traditional analytical models (based on formulas from code, constitutional laws of materials, geometry, and physics), numerical models (such as finite element models), statistical models (based on the correlation between measurable quantities), and sometimes even “black box” algorithms provided by the stakeholders of the asset themselves [67]. Although a Digital Twin can also employ these as its virtual model, the characteristic that distinguishes its application from that of traditional approaches is the previously mentioned property of continuously updating the model on based on structural behavioral and physical property data sampled directly from the Physical Twin.

Note that measuring the response of a structure from its construction stage onward is of critical importance to the development of an accurate Digital Twin model. Indeed, if the monitoring starts during the construction stage, the Digital Twin would include all the structural response variations that occurred during the operational life of the structure, e.g., maintenance work and transit of exceptional loads. In this way, the Digital Twin can accurately reproduce not only the current structural response, but also all its previous history, thus reducing the model uncertainties and improving the accuracy of behavioral predictions. Instead, if the monitoring starts when the structure has already been operational for a number years, the Digital Twin will be limited to being a structural model that is identical to that of the design stage, with input parameters calibrated in such a way that the predicted response matches the measured response.

Because the update process of a Digital Twin is key to its performance, its internal structural model can be considered to be a “deterministic black box” with a set of regularly updated parameters as input and a set of structure response predictions as output. As noted earlier, it is possible to update the Digital Twin in two ways: (1) by directly measuring the model parameters (e.g., concrete strength and Young’s modulus) with in situ tests (e.g., NDTs), and consequently inserting them into the model; or (2) measuring the structural response by means of sensors, comparing it to the model prediction, and indirectly updating the model parameters through an inference process (e.g., Bayesian inference, maximum likelihood estimation, and supervised or even unsupervised learning processes [68]). The former is the most accurate approach but, at the same time, the most taxing and most limited of the two. For this reason, NDTs typically require professional inspectors on site, they are limited in time (the tests cannot be more frequent than one every few months), and the tests—and their consequent data—are limited to the easily accessible part of a structure. In contrast, measuring the structural response of an asset through a sensor network (updating method 2) results in an easier data extraction process, because accurate and objective measurements can be taken by SHM systems in an automated and remote manner. Nevertheless, since the updating of the model is indirect in nature, if great care is not taken when designing both the monitoring system and the updating procedures, this latter method may lead to a higher degree of uncertainty [69]. Focusing only on the second of these updating methodologies, to effectively update a Digital Twin with monitoring observations, the SHM system must be accurately designed.

First, based on the main structural uncertainties of the asset in question, civil engineers must accurately study its static mechanism and identify which of the measurable quantities/parameters are most sensitive to the surpassing of limit states (definition of the EURs). Subsequently, structural operators must define their measurement accuracy to ensure an updated Digital Twin that is precise enough to be used as an informative tool for the proper management of the realized structure. Finally, based on the previous aspects, the monitoring system designer must select the proper sensing technology, with its ensuing performance in terms of measurement accuracy, sampling frequency (or rate), transmission rate, maintainability, measurement range, possibility of integration into a complex monitoring system, etc. [70].

The sampling rate of the sensor network is an especially important aspect to consider. Regarding the turbine example from Tao and Qi [39], the authors efficiently describe the complexity of selecting a sampling rate: “Engineers might monitor vibrations from a turbine gearbox every minute, meaning they would miss shorter glitches. But sampling every second could yield way too much data, leading to transmission bottlenecks”. Once the optimal sensor is selected, the monitoring system designer must define an optimal number of sensors to deploy and their strategic position on the structure [39,71]. Too few sensors may lead to inaccurate descriptions of the assets—on which preventive maintenance relies—and to erroneous predictions, which can work against any process optimization attempt. Too many sensors may also be counterproductive, as the user can be overwhelmed by data, among which key information may be lost—i.e., data dispersion—and the software framework may be taxed excessively, potentially causing bottlenecks, delays, and crashes. The location of the sensor network on the Physical Twin also matters because only its correct positioning allows for the extraction of data comparable to the simulated data extracted from the Digital Twin [59].

To optimize the use of a Digital Twin, it should be updated accurately and frequently. An accurate updating depends mostly on measurement uncertainty (i.e., the sensors’ performance), model uncertainty, and the inference methodology (i.e., the algorithm to update the parameters of the model based on monitoring data). Regarding the latter, several methodologies are now commonly in use. The least-squares deterministic model calibration is an easy and fast inference method; however, this approach strongly suffers from overfitting problems in practical engineering applications, where errors associated with measurements and models are not negligible and datasets are typically too small [68]. Bayesian methods can overcome such a problem by considering the prior distributions of model parameters, which allows outliers in datasets to be neglected (e.g., Strain Reading Anomalies of Distributed Sensing [72]) and prevents a Digital Twin update due to malfunctioning or broken sensors. It should be noted, however, that Bayesian methods typically require an iterative process to estimate the posterior distribution of model parameters (e.g., the Metropolis–Hastings algorithm [73]). Finally, in the past decade, machine learning has experienced rapid growth in its applications to data analysis and numerical model parameter estimations, with both supervised and unsupervised algorithms (the performance of the latter is currently still under investigation) [74].

Generally, the Digital Twin update frequency (also called the refresh rate) depends on the technology (sensors, computational performance of the hardware used), the measured quantity (raw volume of sampled data to process), and the inference method. Different Digital Twin applications require different refresh rates. To elucidate this point, Callcut et al. [1] compared the real-time data requirements for Digital Twins employed in centralized airport air traffic control, in a smart vehicle navigation system, and in a maintenance planning scenario for bridge maintenance. Clearly, the first two applications require real-time refresh rates, whereas the latter does not. As can be implied, a real-time refresh rate is not always possible or necessary. Therefore, it can be stated that a Digital Twin should integrate a new set of attributes and key values only after each significant change in the physical asset, i.e., with frequency analogous to the occurrence of case-to-case “significant” changes [54]. Thus, the best refresh rate is not necessarily real time, but rather “right time” [1]. Note that, in civil engineering, high refresh rates should also be contemplated. This is the case, for example, in structures whose elements are characterized by fragile failure mechanisms (such as shear keys of prestressed concrete bridges) or for structures located in highly seismic areas.

From a practical perspective, the Digital Twin refresh rate depends on the technology, the measured quantity, and the inference method. Regarding the former, if the refresh rate of a Digital Twin is required to match the sampling rate of the sensor network, then it may range from 4000 kH for accelerometers and 250 Hz for DOFS, to one measurement every 15–30 min for robotized topographic stations [22]. Note that the frequency of data transmission can be real time in the case of wired sensors (e.g., Distributed Sensing) or characterized by intervals up to minutes long for wireless sensors (e.g., accelerometers based on the LoRaWAN protocol) [22]. Furthermore, different data may be available at different frequencies because they are acquired by different sensors. In this case, the update process can be instructed to start as soon as different datasets become available or at specific intervals after all the data has been received [75]. Furthermore, depending on the model parameter to be estimated, the analysis can run each time that a new measurement is acquired (e.g., temperature compensation of strain measurements), or it may wait for a dataset (e.g., vibrational frequency estimated through operational modal analysis), thus causing a refresh rate delay. Finally, the analyses may have different durations based on the number of iterations required by the inference method (e.g., one iteration for the least-squares deterministic calibration of a linear model vs. 1000 iterations, at least, for a Bayesian parameter estimation through a Markov Chain Monte Carlo simulation [76]).

Finally, after discussing the Digital Twin inference methodology, the sensor network requirements, and the refresh rate, its operational functionalities are now discussed, with a particular focus on emergency thresholds. Different emergency responses are activated when an “extreme” value is measured or when a previously determined threshold is surpassed. One such approach is described in Ballio et al. [77], who use several deterministic evolution scenarios of the safety conditions of a structure with a clear threshold between them. These scenarios are: Normalcy (no critical situation is foreseen); Alert (situation is evolving to potentially critical); Warning (situation may become critical); and Severe warning (structure is at its limit condition, collapse may happen at any time). The following provides a list of possible threshold-based approaches, based on which the Digital Twin can trigger an emergency alarm and response. These are sorted by response speed and complexity of the involved framework:

- Threshold on measurements. In this scenario, the Digital Twin is updated with monitoring data collected up to instant *t*_−1_, on the basis of which it automatically calculates a predicted structural state or behavior at instant *t* with a specific interval of confidence. Then, the monitoring system measures the real-life structural behavior at time *t* and the software framework compares it against the above predicted behavior. If the measured behavioral value lays outside the upper/lower thresholds of the predicted value’s interval of confidence, the structural behavior is classified as “unexpected” or “divergent”, and an emergency plan is activated [78]. Note that, for this approach, the time requested to activate the alarm depends only on the sampling and transmission frequency of the sensing system. Potentially, this approach allows for a near real-time response to an emergency but is susceptible to false positives and false negatives. As such, it is usually advised to design the emergency response in such a way that the alarm is activated only after observing at least a few similar measurements outside the interval of confidence [79].
- Threshold on the structure condition/health state. The Digital Twin is updated to the last available measurement and the probability of failure of the existing structure is estimated by means of a structural reliability analysis [80]. If the probability of failure is found to be higher than a threshold value (typically around 10^−6^ for civil infrastructure [81]), an emergency plan activates. Here, the time requested to activate the alarm depends on the inference method used to update the model. Deterministic methods operate at close to real time but are less reliable for complex virtual models. Bayesian methods, by comparison, are more reliable but can take up to a few minutes.
- Threshold on the expected utility of management strategies. In this case the Digital Twin is integrated into a Decision Support System. Here, the process of making a decision about the management of a structure is formalized into a decision tree representing the possible management strategies and structural condition states. According to the principles of the expected utility theory or other decision theories [82], decision thresholds can be defined based on the expected utility of management strategies. Monitoring data are then compared to the decision thresholds and an emergency plan is activated accordingly [78]. The time requested to activate the alarm depends only on the sampling and transmission frequency of the sensing system. Precautions must be taken to avoid false positive and negative responses.

The following section outlines the reasons why Distributed Sensing fully meets the requirements specified in the above paragraphs, and thus represents an ideal candidate for the sensor network part of the Digital Twin.

## 5. Distributed Sensing as Digital Twin Sensor Network Component

The application of Distributed Sensing to the civil and structural engineering field has slowly but steadily increased in recent years [23,26], and has gaining increased traction as an SHM tool [19,83]. As testament to this, the IM-SAFE report [15] listed Distributed Sensing among the main trends of the best future practices in monitoring, assessment, and maintenance of transport infrastructure, with a definition as follows: “SHM with novel (non-remote) technologies: distributed sensing, wireless and energy-efficient sensor technologies”.

The reason for this surge in applications can be interpreted in light of OFS features and how these are adapted for SHM purposes [83]. The following lists the DOFS features of interest for such applications:

- DOFS (not all OFS kinds) allow completely distributed monitoring, with monitoring points spaced less than 1 mm apart [25].
- They allow for measurement at high frequencies of 250 Hz [25].
- Their small diameter and minimal stiffness allow for a very high degree of deployment configuration complexity, regardless of whether this implies circumferential surfaces, sharp corners, surface irregularities, etc. It is even possible to embed them inside structural elements with a minimal level of intrusiveness.
- Their ease of deployment can be achieved by simply applying an adhesive over them.
- Their monitoring length is very flexible and can vary from half a millimeter to tens of kilometers.
- They have intrinsic immunity to electro-magnetic interference.
- They are designed with a long life cycle. Indeed, their main component, silica, is highly resistant to corrosion and can withstand high tensile loading.
- Silica core OFS are highly resistant to temperature and can measure temperatures from −200 to 800 °C.

In the following, the authors discuss how, due to these and other Distributed Sensing features, this is the ideal tool to fulfill the role of the sensing component in a Digital Twin.

Potentially, the main goal of a Digital Twin is to provide a clear and accurate picture of the behavior of a structure. As previously mentioned, in order to achieve this goal, it is important to properly select the kind of sensor based on its ability to accurately detect variations in the parameter that has been defined—during the definition of the EURs—as the most indicative of changes in the behavior of the structure. The adjective “accurately” in the previous sentence is notable. This can be understood in different ways, which, nevertheless are all encompassed by Distributed Sensing.

The distributed nature of DOFS measurements enables the mapping of temperature, strain, and vibration distributions at any point along a fiber with a very high spatial resolution (0.63 mm). Consequently, geometrically, Distributed Sensing enables the painting of a very clear picture of the distribution and evolution of these mechanical parameters along the monitored structural elements (versus reporting the tensile state of a limited number of points, as is the case with punctual sensors). This allows the modeling of an accurate digital model inside the Digital Twin that is able to convey comprehensive and accurate information on the state of the monitored structural member. In turn, the availability of this highly detailed data increases the degree of confidence with which infrastructural managers can make a decision and undertake a certain course of action regarding the operative management of their stock of infrastructure.

The comprehensiveness of the data available through DOFS is not restricted to the high spatial resolution, but also encompasses the structural element under surveillance. Indeed, due to their small diameter and minimal stiffness, DOFS can be easily deployed on structural surfaces having limited accessibility and ability to be inspected. Furthermore, an increasingly popular trend in modern research is the embedding of DOFS inside plain or reinforced concrete structures with a minimal level of intrusiveness [19,84]. This provides insight into a structure’s inner workings and physics (e.g., the bond between concrete and steel [85,86]), deformations [87,88], shrinkage [89], deflections [90], and cracking [91]. Due to the internal position of DOFS, the relaying of the above data to the Digital Twin allows for efficient planning of interventions on defective or over-stressed structures, even before any damage appears on their surface.

Distributed Sensing also allows for very frequent measurements (250 Hz). Note that this sampling speed is not reduced by transmission delays because Distributed Sensing falls under the category of “wireless sensors”. Due to this sampling rate, a wide range of possible approaches to Digital Twin updating and emergency planning is available. For example, with a frequency of 250 Hz, one can potentially revalidate the model up to 250 times per second (practically qualifying it as real time) even though, as previously mentioned, it is not necessary that a high sensor sampling rate should be translated 1:1 to a Digital Twin refresh rate. Indeed, this would expose the user to false negatives (and consequent false alarms), overlooking of key clues regarding structural malfunction, and transmission bottlenecks. Nevertheless, a high sampling rate allows swift validation of the correctness of abnormal measurements by comparing a sampled value against several successively sampled values. If it is found that the abnormal measurement is not an outlier but an actual structural behavioral discrepancy (e.g., fragile structural failure or occurrence of an extreme natural event), a high Digital Twin refresh rate may be crucial for a timely trigger of an emergency plan. In short, the high sampling frequency of Distributed Sensing provides the infrastructure manager with a high level of discretion about the choice of the Digital Twin refresh rate, with all options compatible with a wide variety of EURs.

## 6. Conclusions

We live in an environment of ever-growing demand for transport networks, which also have ageing infrastructure. However, it is not feasible to replace all of the infrastructural assets that have surpassed their service lives. The commonly established alternative is to increase their service life by means of Structural Health Monitoring (SHM). Among the multitude of approaches to SHM, the Digital Twin is gaining increasing attention. The present article provides an exploratory review of the key aspects of a Digital Twin, such as its usefulness, modus operandi, and application, and an analysis of the suitability of Distributed Sensing as its sensor network component.

First, the key features of a Digital Twin were identified as follows: (1) the Digital Twin is a virtual representation or replica of a physical real-world asset (i.e., the Physical Twin); (2) in a Digital Twin, the geometric data are enriched by semantic information, engineering data, and operational data extracted from the Physical Twin by means of sensor networks deployed on the latter; (3) the Digital Twin’s biggest departure from traditional digital models is its ability to monitor and report the structural behavior and health of a civil engineering asset throughout the entirety of its service life.

The latter point represents the biggest potential for an SHM application of a Digital Twin. On this topic, the key conclusions were:

- The Digital Twin provides infrastructure managers and decision makers functionalities for controlling, monitoring and, optimizing a physical asset;
- The Digital Twin automatically performs frequent revalidation and updates of the structural model, providing visibility of the evolution of key structural parameters and ensuring that no structural criticality is unnoticed;
- The Digital Twin allows for timely interceptions of sudden differences in the predicted versus measured responses, which are typically symptoms of damage;
- The Digital Twin allows for the proper timing and prioritization of maintenance interventions, thus helping the conversion of the modern time-based maintenance approach to a more performant and sustainable condition-based approach;
- The Digital Twin supports decision makers by providing information on the structural reliability of an infrastructural asset, whether under daily operative conditions or after extreme events such as earthquakes and floods;
- The Digital Twin can help address the skepticism of infrastructural managers in regard to making decisions based only on SHM data;
- The Digital Twin can be integrated into Decision Support Systems and used to consistently support the infrastructural manager in maintaining the operation of an infrastructural asset well beyond its service life;
- The Digital Twin allows for behavioral predictions of an infrastructural asset, which can be used to understand ahead of time whether the structure may transit towards a damaged state (e.g., cracking) or exceed the value for the serviceability limit (e.g., excessive deflection);
- The Digital Twin can allow for a certain degree of operational automation from the structure itself by linking both the Physical and the Digital Twins with a bi-lateral data exchange, effectively establishing a continuous data-feedback loop.

Finally, based on an extended analysis of the key aspects to consider when designing a Digital Twin, the authors assessed the optimal suitability of Distributed Sensing as a sensor network component. The main conclusions were as follow:

- The distributed nature of DOFS measurements enables the mapping of temperature, strain, and vibration distributions at any point along a fiber with a very high spatial resolution (0.63 mm); this allows the construction of an accurate digital model inside the Digital Twin;
- The accuracy of the Digital Twin model made possible by distributed measurements increases the degree of confidence with which infrastructural managers can make decisions and undertake a certain course of action regarding the operative management of their stock of infrastructure;
- Due to their small diameter and minimal stiffness, DOFS can be easily deployed on structural surfaces with limited accessibility and ability to be inspected;
- DOFS can be bonded inside reinforced concrete structures, thus allowing Digital Twins to provide an insight into their inner workings and potential damage; this can allow for efficient maintenance interventions before any damage appears on their surface;
- Distributed Sensing also allows for very frequent measurements (250 Hz), through which the Digital Twin can swiftly validate the correctness of abnormal measurements and, if proven correct (indicative of damage), trigger a timely emergency response;
- The high sampling frequency of Distributed Sensing provides an infrastructure manager with a high level of discretion regarding the choice of the Digital Twin refresh rate; all options are compatible with a wide variety of objectives and management strategies.

In conclusion, the Digital Twin represents a revolutionary step forward for efficient, safe, and sustainable management of civil engineering assets. Furthermore, it was determined that the potential of the Digital Twin can be entirely fulfilled by means of the state-of-the-art monitoring tool, Distributed Sensing.

## Data Availability

Not applicable.
